# Changes in Neurofilament and Microtubule Distribution following Focal Axon Compression

**DOI:** 10.1371/journal.pone.0131617

**Published:** 2015-06-25

**Authors:** Adam J. Fournier, James D. Hogan, Labchan Rajbhandari, Shiva Shrestha, Arun Venkatesan, K. T. Ramesh

**Affiliations:** 1 Mechanical Engineering Department, Johns Hopkins University, Baltimore, MD, United States of America; 2 Hopkins Extreme Materials Institute, Johns Hopkins University, Baltimore, MD, United States of America; 3 Department of Neurology, Johns Hopkins University School of Medicine, Baltimore, MD, United States of America; Stanford University School of Medicine, UNITED STATES

## Abstract

Although a number of cytoskeletal derangements have been described in the setting of traumatic axonal injury (TAI), little is known of early structural changes that may serve to initiate a cascade of further axonal degeneration. Recent work by the authors has examined conformational changes in cytoskeletal constituents of neuronal axons undergoing traumatic axonal injury (TAI) following focal compression through confocal imaging data taken *in vitro* and *in situ*. The present study uses electron microscopy to understand and quantify *in vitro* alterations in the ultrastructural composition of microtubules and neurofilaments within neuronal axons of rats following focal compression. Standard transmission electron microscopy processing methods are used to identify microtubules, while neurofilament identification is performed using antibody labeling through gold nanoparticles. The number, density, and spacing of microtubules and neurofilaments are quantified for specimens in sham Control and Crushed groups with fixation at <1min following load. Our results indicate that the axon caliber dependency known to exist for microtubule and neurofilament metrics extends to axons undergoing TAI, with the exception of neurofilament spacing, which appears to remain constant across all Crushed axon diameters. Confidence interval comparisons between Control and Crushed cytoskeletal measures suggests early changes in the neurofilament spatial distributions within axons undergoing TAI may precede microtubule changes in response to applied loads. This may serve as a trigger for further secondary damage to the axon, representing a key insight into the temporal aspects of cytoskeletal degeneration at the component level, and suggests the rapid removal of neurofilament sidearms as one possible mechanism.

## Introduction

Traumatic brain injury (TBI) and spinal cord injury (SCI) affect over 1.7 million persons in the US annually and results in massive direct and indirect medical costs (over $77 billion in 2010) [1–3]. TBI and SCI can occur through a recognized pathology of traumatic axonal injury (TAI) where rapidly applied mechanical loads lead to changes in the cellular and sub-cellular structure of white matter tracts in the central nervous system (CNS) [4–18]. The load is typically applied at larger length scales in the form of contact, inertia (non-contact), rotational, and blast environments. At smaller length scales, the complex loading environment is understood as a combination of compression, tension, and shear that leads to a series of progressive changes in neuronal axons that heralds the onset of TAI [5, 13–16, 19–21]. While the majority of axons may not undergo immediate disruption (primary axotomy) at the time of loading, the loading leads to a temporal response culminating in a progressive loss of neural connectivity, resulting in physical and cognitive disability (secondary axotomy) [20, 22–24]. One of the key features first observed in the progression of secondary axotomy are morphological and structural changes associated with disruption of the cytoskeletal network [13–18, 20–23].

The cytoskeleton is an elaborate network of proteins, having a variety of configurations, whose purpose is to provide functional and structural stability to the cell. Major constituents of the cytoskeleton in CNS axons include neurofilaments (NFs) and microtubules (MTs). Neurofilaments provide mechanical strength, mechanical stability, and determine axon diameter, whereas microtubule functions include intracellular transport and provision of structural rigidity [25–28]. Neurofilaments are heteropolymers that consist of three polarized sidearm subunits: NF-light (~70kDa), NF-medium (~150kDa), and NF-heavy (~200kDa). The subunits attach to a core measuring approximately 12nm in diameter, and the polarized sidearms function to space neurofilaments from each other at regular intervals across the axon diameter [25, 26]. Microtubules are composed of α- and β-tubulin heterodimer subunits assembled into linear protofilaments, have an approximate molecular weight of 50kDa, and form as 24nm wide hollow cylinders [27, 28]. Secondary axotomy is linked to the disruption of microtubule and neurofilament networks in white matter tracts of the CNS (as a result of mechanical perturbation) and is associated with the formation of nodal blebs, or swellings, along the length of the axon at the site of injury [7, 13–17, 21–23].

*In vitro* approaches to understanding axonal injury include models based on transection, shear, compression, hydrostatic pressure, hydrodynamic changes, acceleration, and cell stretch [29]. By designing controlled *in vitro* models, researchers are able to study how the mechanical insult applied can influence the acute mechanisms of axonal injury, where the aim is to mimic the structural consequences of the injury rather than replicating the mechanical loading conditions observed at larger length scales. Pettus and Povlishock qualitatively provide evidence of microtubule disruption and neurofilament compaction within 5min of fluid percussive injury using electron microscopy [30]. This indicates changes in the cytoskeletal structure can be observed at short time intervals following loading. Quantification of changes in microtubule and neurofilament expression following secondary axotomy has been accomplished through a variety of animal models [5, 7, 13, 15–17, 19, 21, 23, 30, 31]. Researchers have quantified the number, spacing, and densities of microtubules and neurofilaments following stretch injury to guinea pig optic nerves and indicate a loss in cytoskeletal components as early as 15min following load [13, 15, 16, 21]. While these studies provide insight into changes in cytoskeletal structure following TAI and its temporal evolution, quantifying the immediate changes in cytoskeletal distribution remain unknown.

The complex character of fiber families, fiber orientations, and fiber tract distributions in the brain ensure that macroscopic insults to the head result in the full gamut of deformation states at the axonal scale, including local tension, focal compression, kinking, and shear. Although the response to tension has been well studied, the consequences of focal axon compression are poorly understood and this state is considered in this paper.

Our lab has recently demonstrated visualization and quantification of three-dimensional (3D) live subcellular populations under controlled focal compression using a confocal microscope at high magnification, where transmission electron microscopy (TEM) of fixed samples was utilized to corroborate the observed confocal changes with greater spatial resolution [32]. Our results suggest changes at the substructure level of neurofilaments may precede microtubule rupture and degeneration for CNS axons undergoing TAI in response to applied mechanical loads [32]. In the current study, we quantify changes in NFs and MTs in the setting of focal compression through the use of TEM in order to develop insights as to how cytoskeletal populations change immediately following loading (fixation <1min), how these results fit into previously conducted research, and how this information might be effectively utilized towards the development of an analytical model for understanding the changes in cytoskeletal constituents following TAI over time.

## Methodology

### Axon Injury Micro-Compression Platform

A previously designed axon injury micro-compression (AIM) platform is utilized for this study [19]. The platform is composed of polydimethylsiloxane (PDMS) (Sylgard 184; Dow Corning; Midland, MI, USA) and is mounted to a cleaned 50mm #1 glass-bottom petri dish (Wilco Wells; Amsterdam, The Netherlands). The AIM platform consists of two levels: a flow layer and a control layer. The flow layer contains a series of three compartmental chambers for neuronal cell bodies, proximal, and distal axons. The control layer provides a microfluidic pressure system for applying focal compression loads to segments of axons. The AIM platform construction process followed established protocols and involves standard 10:1 base to cross-linker ratios by mass for the PDMS [19].

The flow layer master template, where the neurons and media reside, is made from three aligned and stacked layers of silicon wafers (WRS Materials; San Jose, CA, USA) processed with 3005 and 3025 SU-8 (Microchem; Newton, MA, USA). Once the master template is developed, the mold is spin-coated with a thick layer of PDMS pre-polymer and fully cured at 80°C for 20mins.

The control layer master template, for controlling the injury pad, is patterned using a silicon wafer and 3050 SU-8 (Microchem; Newton, MA, USA) and is developed with a thick layer of PDMS. Once cured, individual devices are cut, and access ports for control fluidics are punched using sharpened gauge #23 needles (McMaster-Carr; Santa Fe Springs, CA, USA).

PDMS castings from both template layers are exposed to an oxygen plasma cleaner (Harrick Plasma; Ithaca, NY, USA) and surface treated (30 Watts; 1.5mins). Injury pad control layers are visually aligned with the flow layer features, brought into intimate contact, and baked overnight at 80°C to amalgamate PDMS layers. Composite devices are removed and access ports are created using a 3mm biopsy punch tool (Huot Instruments; Menomonee Falls, WI, USA). Devices are sterilized by ethanol sonication, autoclaved, and sealed to a cleaned 50mm #1 glass-bottom petri dish (Wilco Wells; Amsterdam, The Netherlands) prior to use.

A 100μg/mL solution of Poly-D-Lysine (Sigma; St. Louis, MO, USA), an extra cellular matrix that facilitates axonal growth diluted in molecular grade water (Mediatech; Herndon, VA, USA), is introduced by way of access ports in the platform. The AIM devices are incubated overnight at 37°C in a humidified, 5% CO_2_ incubator. The following day, devices are washed 3x with double deionized water (ddH_2_O) to remove unbound PDL and neurobasal media (Gibco Life Technologies; Grand Island, NY, USA) is added for cells to be seeded.

### Cell Culture and Isolation

Primary hippocampal neurons are isolated from E17 Sprague Dawley rat pups (Charles River, Wilmington, MA, USA) following the specific regulations approved by the Johns Hopkins Institutional Animal Care and Use Committee (protocol RA12M213). Dissociated neurons are loaded into the somal compartment at a density of 25x10^6^ cells/mL in a mixture of neurobasal media (Gibco Life Technologies; Grand Island, NY, USA), penicillin-streptomycin (Life Technologies; Grand Island, NY, USA), B27 (Life Technologies; Grand Island, NY, USA), hepes (Gibco Life Technologies; Grand Island, NY, USA), and L-Glutamine (Life Technologies; Grand Island, NY, USA). After a period of 6–8 days in culture, axons can be observed extending into the middle and distal chambers of the AIM device in sparse numbers to allow tracking of individual processes for subsequent experiments. Media composed of neurobasal media (Gibco Life Technologies; Grand Island, NY, USA), penicillin-streptomycin (Life Technologies; Grand Island, NY, USA), B27 (Life Technologies; Grand Island, NY, USA), and hepes (Gibco Life Technologies; Grand Island, NY, USA) is added every 3 to 4 days to maintain neuronal viability.

### Load Application

As reported in previous studies, dynamic compression is applied exclusively to axons of primary hippocampal neurons [19]. Four independent tubing lines (O.D. = 1.52mm, I.D. = 0.51mm; Cole Parmer; IL) attaching to gauge #21 blunt needles (McMaster-Carr; Santa Fe Springs, CA, USA) are coupled to each of the four control fluidic ports of the AIM prior to loading. The control gas (CO_2_) pressure is manipulated with a Proportion Air electronic regulator (Equilibar; Fletcher, NC, USA) to apply the desired level of pressure between the glass substrate and injury pad.

Using a validated finite element model, geometric parameters for each platform provide the input fluidic pressure required to obtain the injury response observed in TAI [19]. Pressure application is performed by adjusting the electronic pressure regulator, turning the stopcock ninety degrees to allow pressure translation to the injury pad (<1s), holding the pressure (<5s), and relieving the pressure by turning the stopcock back to the start position. Live imaging is used to estimate the time scale for dynamic compression to be on the order of 1.5ms for axons with a range of diameters from 0.25–2.0μm as observed through confocal and TEM data [32]. Visual confirmation of contact between the compression pad and glass substrate is observed for all groups during load application. Fix is immediately added through access ports following loading and tubing connectors are removed. Following a period of 15min in fix, AIM platforms are peeled from the glass bottom petri dishes and additional fix is added to the dish. Sham controls are prepared and data is recorded in the same manner as those undergoing dynamic compression, but no loading is applied.

### Fixation, Labeling, and Embedding for TEM

Different approaches are used for preparing microtubule and neurofilament TEM specimens. The choice of approach is made by identifying what each group requires in terms of morphology and labeling. In our study microtubules are relatively easy to identify and quantify, so morphology is of paramount concern. Neurofilaments, by comparison, are harder to identify and require immuno electron microscopy with gold (Au) labeling using 2°NF-medium antibodies to confirm presence within processed TEM sections. Previous research regarding immunogold labeling using antibody specific binding has been shown to be effective in detecting the presence of a specified molecule of interest [33–35].

One inherent limitation of using immunogold labeling is that the observed Au-nanoparticle is approximately 15–30nm from the primary binding site of the antibody [36]. However, the spacing of the nanoparticles from their binding sites should be comparable and the number density of the nanoparticles is equal to the number density of the binding sites. As labeling is of primary importance for the neurofilaments, the quality of morphology determination is sacrificed in the TEM images in favor of labeling.

Microtubule TEM samples are fixed with a mixture of 2% paraformaldehyde, 2.5% glutaraldehyde, 0.1M sodium cacodylate (SC), and 1% sucrose for 1hr. Cells are washed with a 0.1M SC, 3mM calcium chloride (CaCl_2_), and 3% sucrose buffer in three 10min rinses and stored in 1% osmium plus 0.8% potassium ferricyanide on ice and in the dark for 1hr, followed with three 0.1M maleate buffer rinses, 5min each. Cells are stained with 2% aqueous uranyl acetate (0.22μm filtered, 1hr, dark) in 0.1M maleate buffer. Cells are dehydrated in a graded series of ethanol washes: 30%, 50%, 70%, 90% and 100% with 10min between washes.

Neurofilament TEM samples are fixed with a mixture of 6% paraformaldehyde, 0.5M SC, and 1.0M CaCl_2_ for 1hr, washed with a 0.1M SC buffer in three 10min rinses and blocked with 1% BSA at 4°C for 1hr. BSA is removed and a mixture of the primary NF-medium antibody (CN: ADI-NBA-140-E, Enzo Life Sciences, Farmingdale, NY, USA) and 0.02% saponin in 0.1M SC buffer (1:100) is added, and cells are incubated overnight at 4°C. Cells are incubated back to room temperature and washed with 0.1M SC buffer six times in 10min rinses. A secondary antibody mixture of 6nm Au particles (CN: 703-195-155, Jackson Immuno Research Laboratories, Inc., West Grove, PA, USA) and 0.01% saponin in 0.1M SC buffer (1:200) is added, and the cells are incubated at 4°C for 4hrs. Cells are incubated back to room temperature for 10min, rinsed with 0.1M SC buffer six times with 10min between rinses, and then 1% glutaraldehyde is added in 0.1M SC for 1hr. Cells are rinsed three times with ddH_2_O, with 10mins between rinses. 0.5% osmium tetraoxide in 0.1M SC buffer is added and cells are placed on ice and in the dark for 30min. Cells are rinsed with ddH_2_O 3 times, with 10mins between rinses, followed by dehydration in a graded series of ethanol washes: 30%, 50%, 70%, 90% and 100% with 10min between washes.

Following the graded dehydration by ethanol, cells for both microtubule and neurofilament samples are embedded in a resin of Epon using DMP-30 as a catalyst and incubated overnight at room temperature. Cells go through a series of Epon + DMP-30 changes and are subjected to vacuum in an attempt to thoroughly embed cells in the resin. Cells are incubated overnight at 65°C. Cells are separated from the dishes through a process of thermal shocking, by placing samples into liquid N_2_, and embedded specimens are retrieved for post-embedding processing.

Samples are cut down to specified grids and go through a triple staining process with 1% tannic acid (aqueous) (Mallinckrodt Pharmaceuticals, St. Louis, MO, USA), filtered using 0.22μm filter, for 10min by flotation using formvar coated slot grids (Ted Pella, Inc., Redding, CA, USA) before being rinsed with ddH_2_O for one minute. Grids are then stained with aqueous 2% uranyl acetate (Polysciences, Inc., Warrington, PA, USA) for 20min and rinsed with ddH_2_O. Finally grids are stained for 1min on 0.04% lead citrate (aqueous-filtered) and rinsed with ddH_2_O. Longitudinal and transverse TEM slices (60–80nm thickness) are obtained and serial sections are taken to address concerns of embedding and processing 3D embedded substructures [37].

### Substructure Quantification Using TEM and Image Analysis

**Fig 1:**
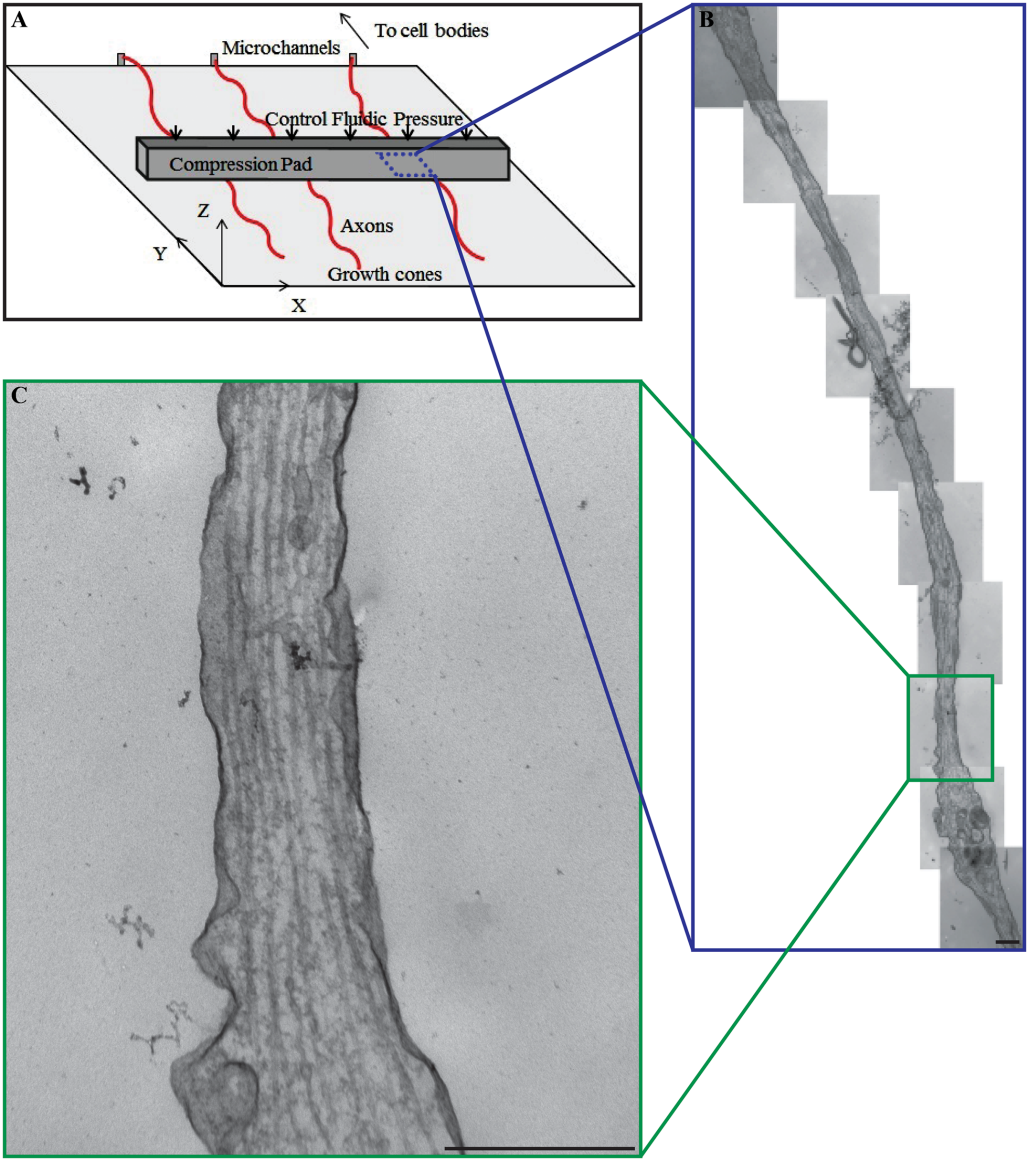
Focal compression of isolated primary hippocampal axons. (A) Schematic illustration of axon loading environment and orientation within the AIM. The axon only region overlaps with a 20μm thick compression pad above the testing chamber. Microfluidics are used to control the compression pad and localize loading to the area underneath the pad (blue box). (B) A series of panoramic TEM images reconstructing the entire area of the axon under the compression pad at higher magnification. (C) A single TEM image for quantifying number, density, and spacing of the cytoskeletal structures. Scale bar = 500nm.

Our TEM measurements for substructure quantification utilize a series of longitudinal sections taken at the same magnification (x46,000) and examine the entire segment under the compression pad (Fig 1). Previous researchers have used transverse examination of axons as a method for examining changes in cytoskeletal structure following TAI [15, 38]. However, given the loading methodology employed in this study and the heterogeneous distribution of cytoskeletal components observed by previous researchers in cross sections of TEM axon samples, the authors found it more useful to examine the longitudinal section of the entire length of axons under the compression pad [15, 38].

Quantification of TEM images requires metrics for assessing quantity, spacing, and density. Previous researchers indicate a relationship between the axon diameter and cytoskeletal measures within axons [15, 16]. Therefore, our TEM results are segmented by diameter to assess required measures at known diameters.

For both microtubule and neurofilament groups, 15–20 axons were examined for each Control and Crushed axon subgroup. A minimum of 10 TEM images were required to construct a single axon spanning the area of the compression pad and 3 measurements of number, density, and spacing were taken from each image. The combined data resulted in 450–600 measurements for Control and Crushed axons of microtubules and neurofilaments groups.

For microtubules, the length of the axon (*L*) for a given TEM image is measured and then divided into quarters. Axon diameter (*D*) and the number of MTs (*N*_*MT*_) are measured at L/4, L/2, and 3L/4. The microtubule linear density across the diameter of the axon ($\rho_{L_{MT}}$) is calculated from the number of MTs and the axon diameter:

$$\rho_{L_{MT}}=\frac{N_{MT}}{D}$$ (1)

The average spacing for microtubules (*S*_*MT*_) at a given *D* is then related to the linear density and corrects for the non-zero thickness (*t*) of the microtubules:

$$S_{MT}=\frac{D-N_{MT}\cdot t}{N_{MT}}=\frac{1}{\rho_{L_{MT}}}-t$$ (2)

In contrast to the microtubule measures that can use linear metrics, neurofilament measurements depend on the quantification of the Au-nanoparticles over a specified area. The neurofilament measurements are made by dividing the axonal length into thirds for a given TEM image and measuring the diameter at the midpoint of each of these thirds. For each of the thirds the areal density ($\rho_{A_{NF}}$) of Au-nanoparticles, defined by

$$\rho_{A_{NF}}=\frac{N_{NF}}{A}$$ (3)

is computed measuring the number of 6nm Au-nanoparticles (*N*_*NF*_) and the area of the axon (*A*) using a code written in MATLAB (Mathworks; Natick MA, USA):

The average spacing of NF (*S*_*NF*_) is computed from the average distance between a given Au-nanoparticle and its nearest Au-nanoparticle neighbor. The spacing is measured for all Au-nanoparticles in a given sample and assumes a proportional binding relationship where the observed Au-nanoparticles requires the presence of the NF-medium binding sites and infers the existence of NFs.

### Statistical Analysis of Data

The collected data is divided into four groups: (1) microtubule Control cells, (2) neurofilament Control cells, (3) microtubule Crushed cells, (4) neurofilament Crushed cells. The data is further separated into bins covering the ranges of axon diameters used: <0.5μm, 0.5–1.0μm, 1.0–1.5μm, and 1.5–2.0μm to evaluate the relationship between axon caliber and cytoskeletal measures. Bonferroni’s multiple comparison tests are used to determine the significance (p < 0.05) between Control and Crushed groups, for a specific axon diameter bin.

## Results

### Morphological Assessment of Microtubules

**Fig 2:**
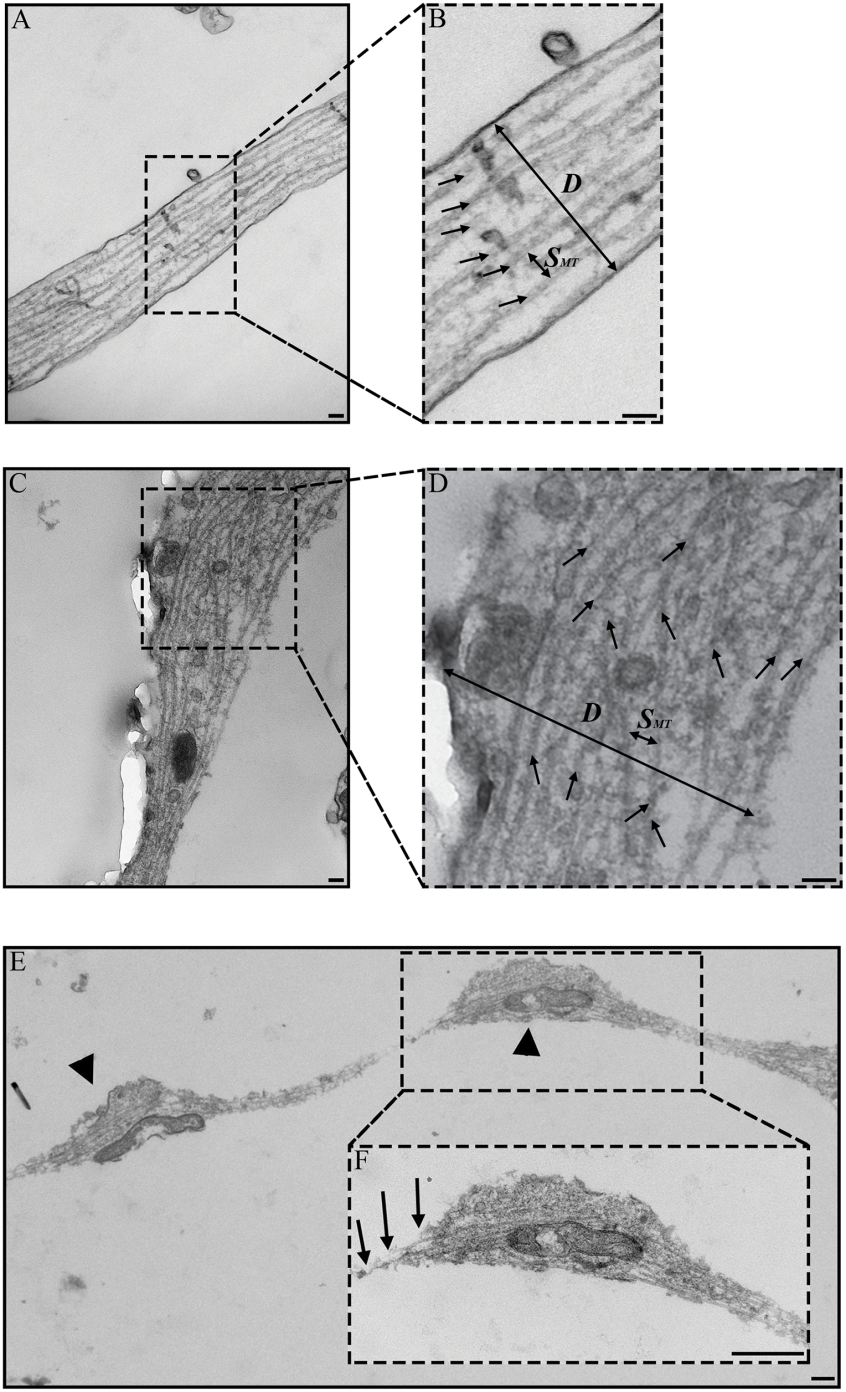
Representative TEM images of microtubules in axons. Microtubules are indicated by black arrows (B, D). Axon diameter, number of microtubules, and spacing between microtubules were measured for each image along unit axon length, L, at L/4, L/2, and 3L/4. (A) In Control axons, microtubules are oriented along the principal axis of the axon. (C) In Crushed axons, microtubules appear disorganized and misaligned. (B, D) Inset of Control and Crushed axons showing diameter (***D***) and spacing (***S***_***MT***_) measurements for microtubules. (E) Nodal bleb (arrow head) of Crushed axon showing mitochondria in each bleb. (F) Inset of Crush axon showing microtubule breakage and rupture (arrows). Parts of fig are adapted from [32]. Image used with permission from the Federation of American Societies for Experimental Biology or The FASEB Journal (www.fasebj.org). Scale bars = 100nm (A-D); 500nm (E-F).

Microtubules are identifiable as long rod-like structures ~24nm in diameter within the axon (Fig 2). For the Control group, microtubules are observed along the primary axis of the axon and regularly distributed across the axon diameter (Fig 2A–2B). For the Crushed group, microtubules appeared to be unchanged in areas where no nodal blebs or swellings are evident (Fig 2C lower center). In areas where swellings are present, the microtubules (arrows) on average aligned along the axis of the axon; however the distribution is frayed and disorganized (Fig 2C upper right, Fig 2D). Microtubules of the Crushed group exhibit breaking points (Fig 2E–2F) where the ends appear to be disjointed (arrows) within nodal blebs (arrow heads). Spatially, the nodal blebs appeared restricted to the compressed axon volume and the portions of the axon immediately adjacent to this volume.

### Quantitative Assessment of Microtubules

**Fig 3:**
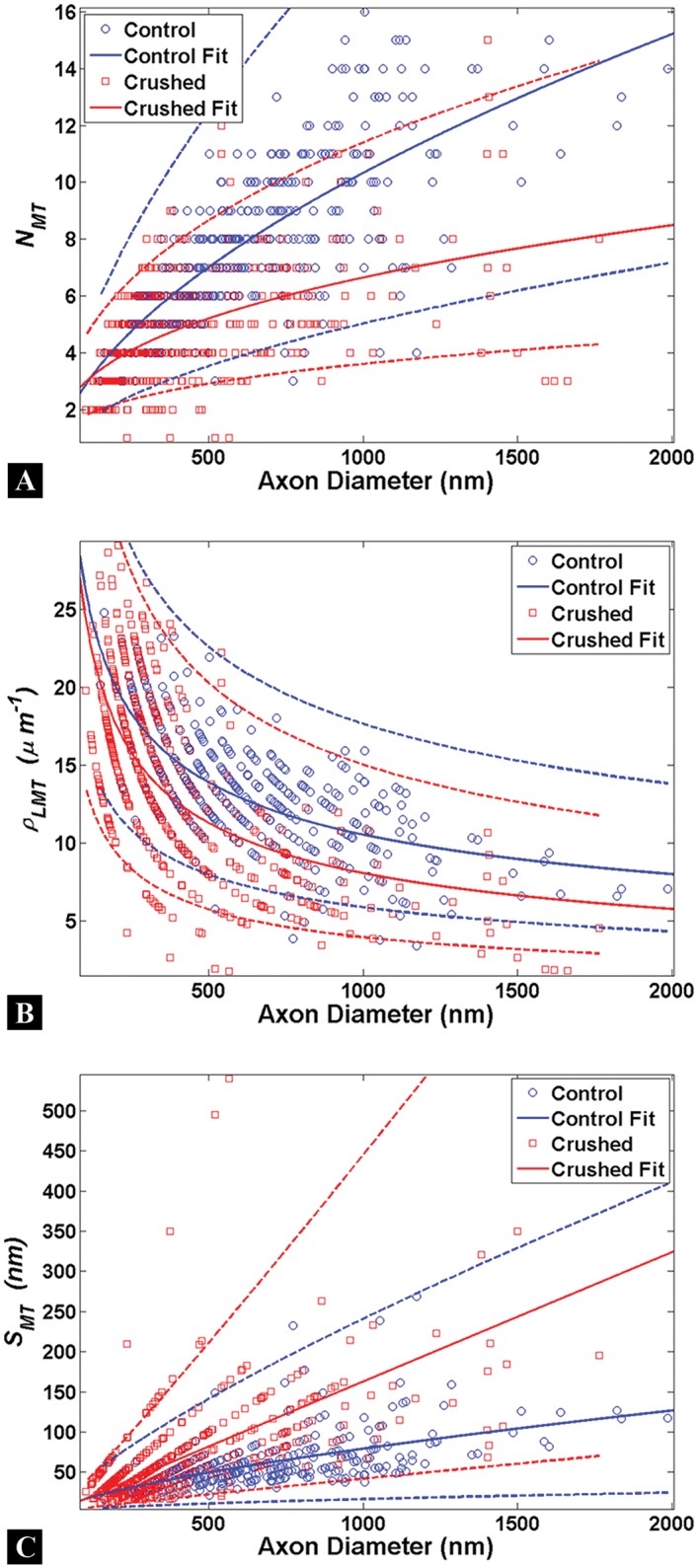
Microtubule quantification in axons. Each circle represents a single measure for the Control (blue) or Crushed (red) groups. Power law fits (solid lines) are shown to estimate the relationship between axon diameter and microtubule metrics with 95% confidence intervals (dashed lines). (A-C) A strong dependency on axon caliber is observed for all microtubule measures for Control and Crushed axons; however the populations of Control and Crush axons are not distinguishable given the overlap of the confidence intervals between both groups. The smallest diameters measured are approximately 100nm and no data was taken for axons of a smaller caliber than this.

The number (*N*_*MT*_), linear density ($\rho_{L_{MT}}$), and spacing of (*S*_*MT*_) microtubules are the quantitative measures taken from axons fixed <1min after loading and compared for Control and Crushed groups (Fig 3A–3C). Previous researchers indicate the number, spacing, and density of microtubules in control axons to be measures with a strong linear dependency on axon caliber [13, 15, 16, 21]. Our results for these measures are shown in Fig 3A–3C with 95% confidence intervals. Note that the data points in Fig 3A–3C appear in bands only because the values of *N*_*MT*_ in Fig 3A must be integers. The dependency of the microtubule number on axon diameter is described well by a power law fit in the form

$$N_{MT}=aD^{m}$$ (4)

where *a* and *m* are fitting parameters for the power law. The power law fits work well for the majority of the data population, but is less reliable at the outliers (for axons where *N*_*MT*_< 3 or *N*_*MT*_ >15). S1 Table provides the coefficients for the power law fits for each of the metrics shown in Fig 3A–3C.

It follows that the microtubule linear density and spacing go as

$$\rho_{L_{MT}}=\frac{N_{MT}}{D}=aD^{m-1}$$ (5)

and

$$S_{MT}=\frac{D-N_{MT}\cdot t}{N_{MT}}=\frac{D^{1-m}}{a}-t$$ (6)

The parameters are shown in S1 Table for both Control and Crushed axons.

**Fig 4:**
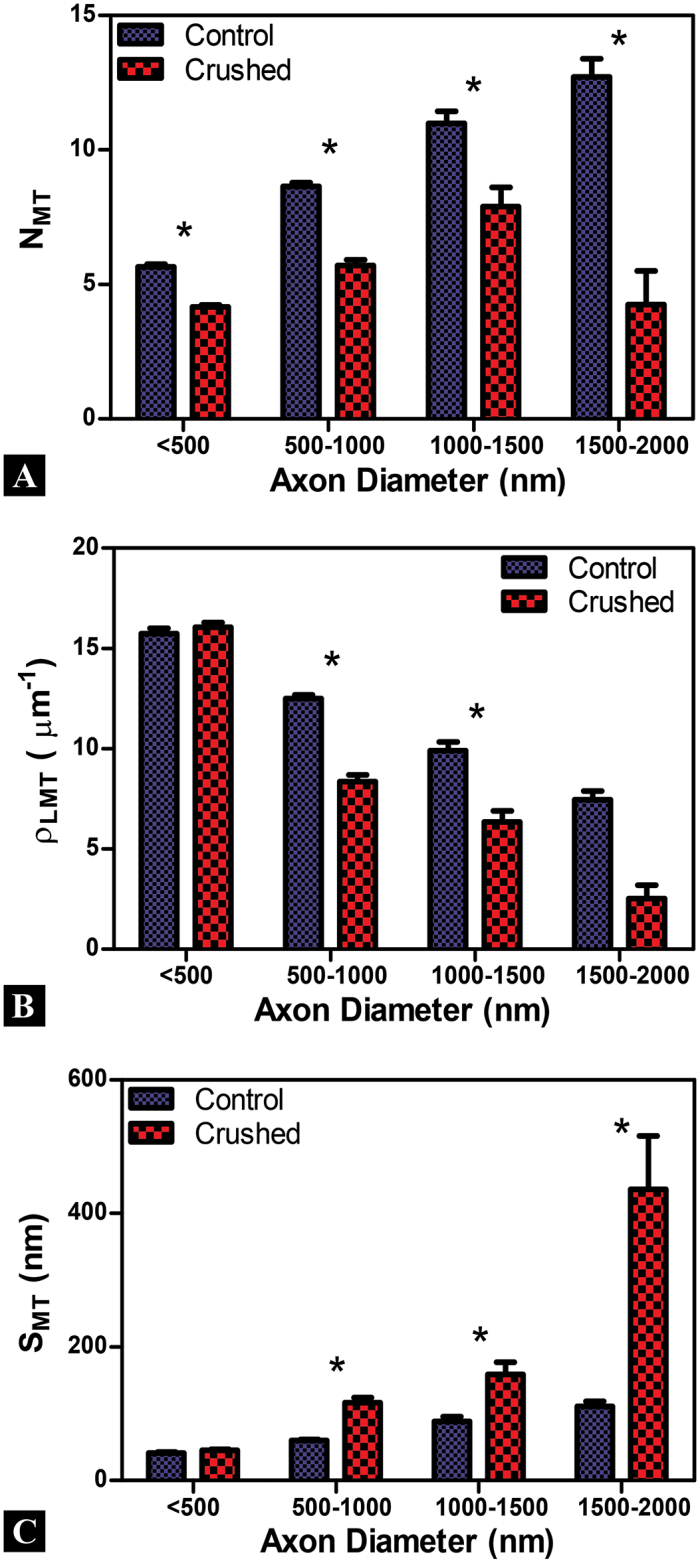
Examination of binned microtubule data. Axon diameter bins are given along the X-axis and error bars are standard error mean for all plots. (A-C) A strong dependency on axon caliber is observed for the number of (*N*_*MT*_), linear density ($\rho_{L_{MT}}$), and spacing between (*S*_*MT*_) microtubules in Control and Crushed axons. *N*_*MT*_ and $\rho_{L_{MT}}$ are significantly lower for Crushed, while *S*_*MT*_ is significantly higher for Crushed, across nearly all axon diameter. The observed differences between Control and Crushed are more apparent at larger axon diameters. *(p<0.05)

When the raw data is binned by axon caliber into 500nm demarcations, an approach frequently used in the literature, the contrasts between Control and Crushed groups for microtubule measures are readily apparent (Fig 4) [13, 15, 16, 21]. *N*_*MT*_ increases with axon diameter in the Control group (Fig 4A) [16]. Similarly the decrease in $\rho_{L_{MT}}$ and increase in *S*_*MT*_ for Controls has been previously noted as the diameter of the axon increases (Fig 4B–4C) [13, 15, 16, 21].

The *N*_*MT*_ for the Control group is significantly higher than the Crushed group across all axon diameters (p<0.05) (Fig 4A). The $\rho_{L_{MT}}$ for the Control group is higher than the Crushed group, though the result is statistically significant only for 0.5–1.5μm diameter axons (Fig 4B). The *S*_*MT*_ for Crushed axons is significantly higher than the Control groups at axon diameters of 0.5μm and above, indicating that the microtubules may be spreading out in response to the applied load (Fig 4C). A common observation was that the differences between Control and Crushed groups are more apparent at the larger axonal diameters for all measures taken.

The mean number of (${\overset{-}{N}}_{MT}$), linear density (${\overset{-}{\rho }}_{L_{MT}}$), and spacing between (${\overset{-}{S}}_{MT}$) microtubules is presented for Control and Crushed groups for all axon bin diameters in Table 1. While we explored a focal compression injury model, similar changes in microtubule parameters have been seen by previous researchers (who conducted tensile loading of axons to induce TAI) where ${\overset{-}{N}}_{MT}$ and ${\overset{-}{\rho }}_{L_{MT}}$ was decreased for loaded (Crushed) axons than Control axons for the same axon caliber [15, 16, 21]. Also, the increased ${\overset{-}{S}}_{MT}$ observed following tensile loading for loaded (Crushed) axons is seen in our data [15, 16, 21].

**Table 1:** Quantification of microtubules and neurofilaments following focal compression.

*Cytoskeletal Measurement*	*Control ± SEM* ^1^	*Crushed ± SEM* ^1^	% *Change from Control ± SEM* ^1^
Mean MT Number ${\overset{-}{N}}_{MT}$	9 ± 2	6 ± 1	-33 ± 10
Mean MT Linear Density ${\overset{-}{\rho }}_{L_{MT}}$ (μm^-1^)	11 ± 2	8 ± 3	-27 ± 13
Mean MT Spacing ${\overset{-}{S}}_{MT}$ (nm)	75 ± 15	189 ± 27	152 ± 62
Mean NF Number ${\overset{-}{N}}_{NF}$	91 ± 36	40 ± 13	-56 ± 22
Mean NF Areal Density ${\overset{-}{\rho }}_{A_{NF}}$ (μm^-2^)	46 ± 14	25 ± 6	-47 ± 21
Mean NF Spacing ${\overset{-}{S}}_{NF}$ (nm)	55 ± 8	70 ± 3	28 ± 20

^1^ Standard error mean (SEM) where n is the number of 500nm demarcated bins for axon caliber. Microtubules (n = 4) and neurofilaments (n = 3) for Control and Crushed populations.

### Morphological Assessment of Neurofilaments

**Fig 5:**
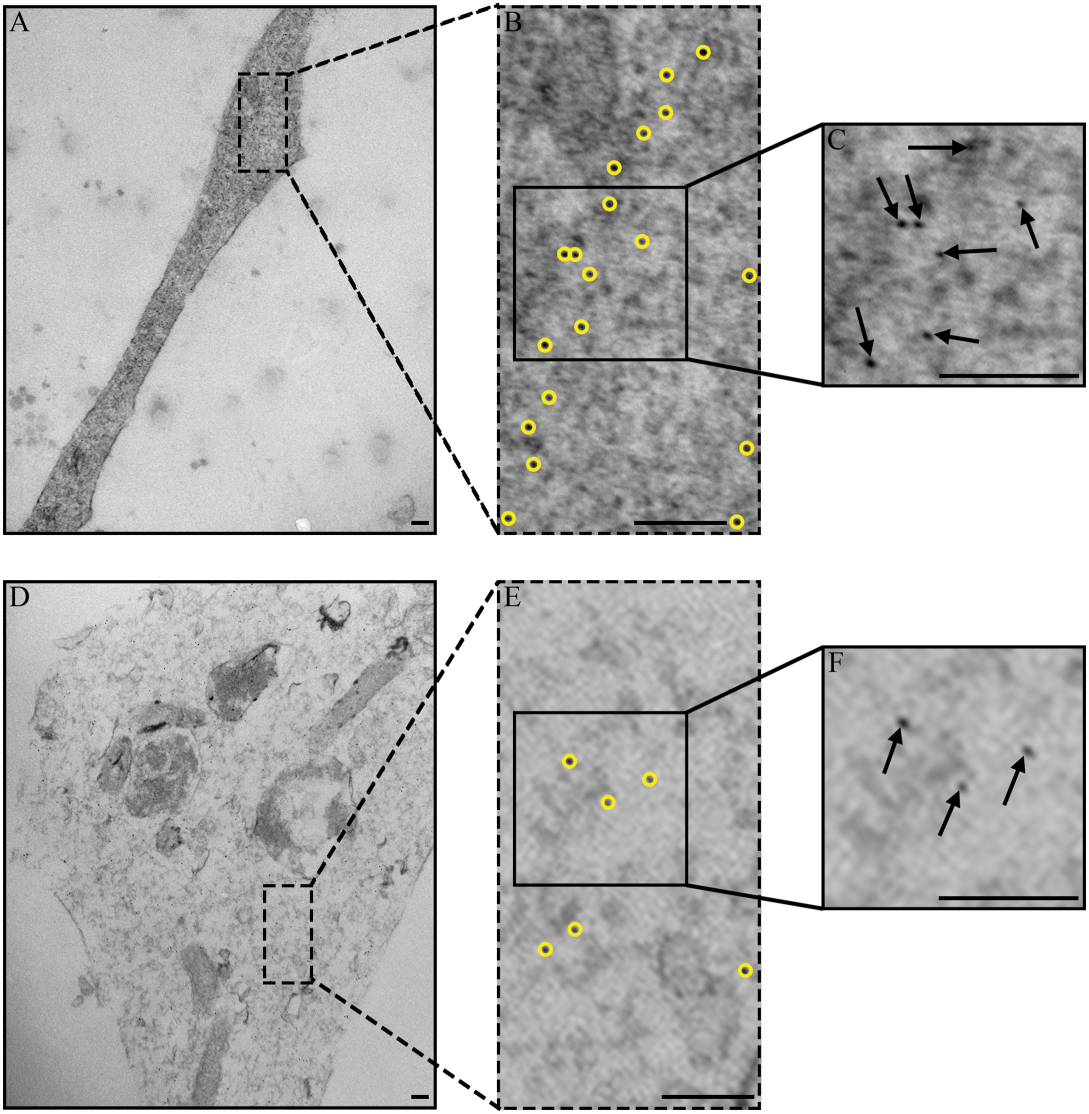
Representative TEM images of neurofilaments in axons. 6nm Au-nanoparticles, outlined in yellow, were used to measure areal density and spacing between neurofilaments for each image. (A-C) In Control axons, Au-nanoparticles are regularly spaced along the length of the axon and across the axon diameter. (D-F) In Crushed axons, Au-nanoparticles appear more heterogeneous in their distribution and spacing. (B-C, E-F) Insets of Control and Crushed axons showing areal density and spacing distribution for nanoparticles. There is a greater number and areal density in Control axons (C) than in Crushed axons (F). Scale bars = 100nm.

Neurofilaments (localization confirmed by Au labeling using 2°NF-medium antibodies) are identified within treated axons (Fig 5). In these examples, the 6nm Au-nanoparticles are highlighted with yellow circles (Fig 5B and 5E). Insets of Fig 5 at higher magnification show the nanoparticles as small black dots (arrows) distributed within the (Fig 5C and 5F). In the Control group, Au nanoparticles are spaced regularly along the length of the axon and across the axon diameter indicating neurofilaments are distributed uniformly within the axon (Fig 5A–5C). The number and areal density of the Au-nanoparticles for the Crushed group appears lower than the Control group and the spacing of Au-nanoparticles is heterogeneous within the axon membrane in areas with and without nodal blebs. This indicates that the sidearms governing the neurofilament distribution have been modified by the loading (Fig 5D–5F).

### Quantitative Assessment of Neurofilaments

**Fig 6:**
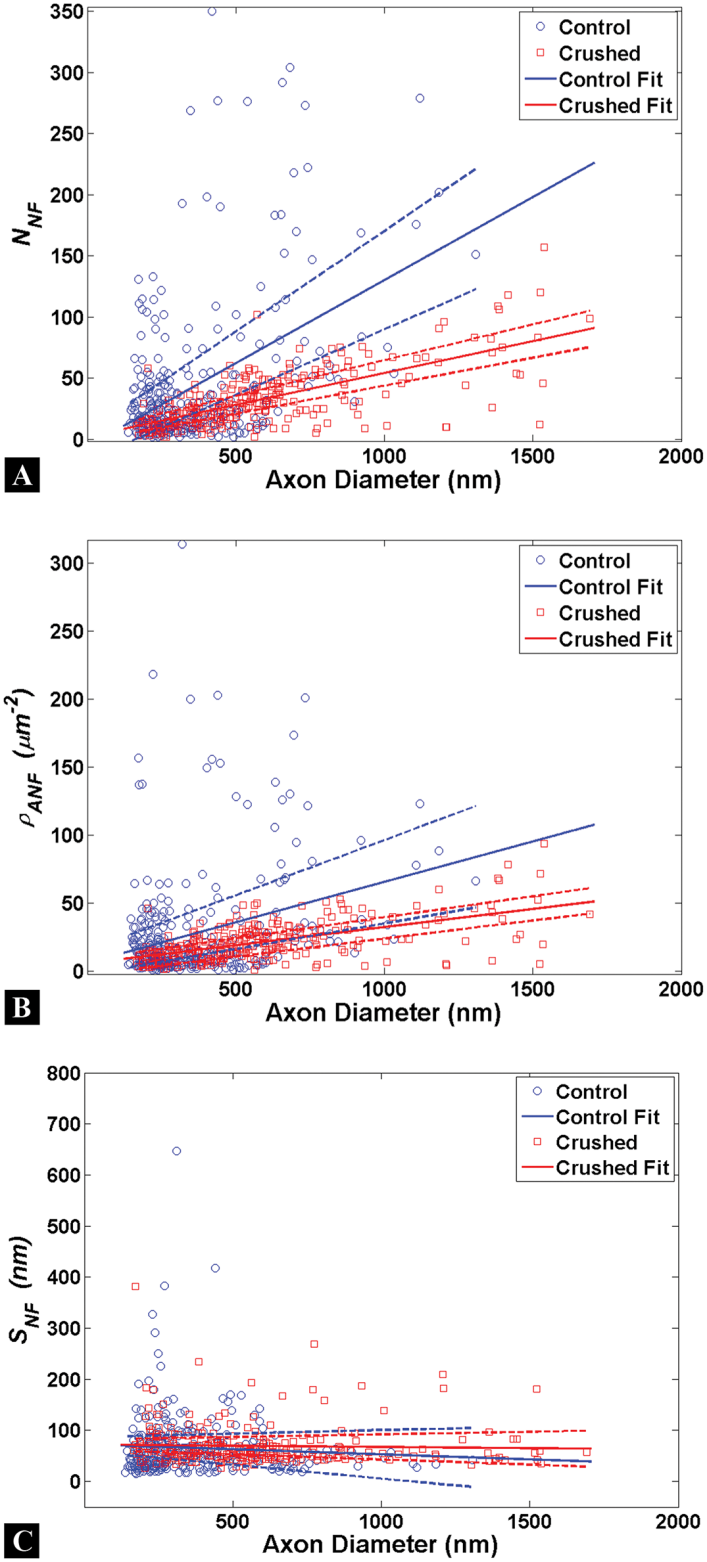
Neurofilament quantification in axons. Each circle represents a single measure for the Control (blue) or Crushed (red) groups. Linear fits (solid lines) are shown to estimate the relationship between axon diameter and neurofilament metrics with 95% confidence intervals (dashed lines). (A-C) A strong dependency on axon caliber is observed for almost all neurofilament measures in Control and Crushed axons; the exception being neurofilament spacing for Crushed axons. The populations for Control and Crushed axons can be separated more readily for *N*_*NF*_ and $\rho_{A_{NF}}$ at larger axon calibers; however *S*_*NF*_ populations are not distinguishable given the confidence interval overlap between Control and Crushed groups. No data was taken for axons of a caliber <100nm; therefore our linear fit is limited in applicability to the data range shown.

The number (*N*_*NF*_), areal density ($\rho_{A_{NF}}$), and spacing of (*S*_*NF*_) neurofilaments (identified by Au-nanoparticles) are the quantitative measures taken and compared for Control and Crushed neurofilament groups (Fig 6A–6C). Note that no Control axons were found having an axon diameter 1.5–2.0μm for this assessment. Previous work has shown that the number, spacing, and areal density of neurofilaments in control axons are measures with a strong dependency on axon caliber [13, 15, 16, 21].

Our results, in terms of raw data, are shown in Fig 6A–6C with 95% confidence intervals. The data in Fig 6A–6C does not exhibit the same power law data bands observed for the microtubules in Fig 3A–3C. However the populations can be distinguished using a linear fit in the following form

$$y(D)=p_{1}D+p_{2}$$ (7)

where y represents the cytoskeletal measure, *D* is axon diameter, and *p*_1_ and *p*_2_ are linear fitting parameters. The linear fits work well for the majority of the data population, but becomes less reliable at the outliers of the data set (for axons where *N*_*NF*_< 10 or *N*_*NF*_ >100). S2 Table provides the coefficients for the linear fits for each of the metrics shown in Fig 6A–6C.

**Fig 7:**
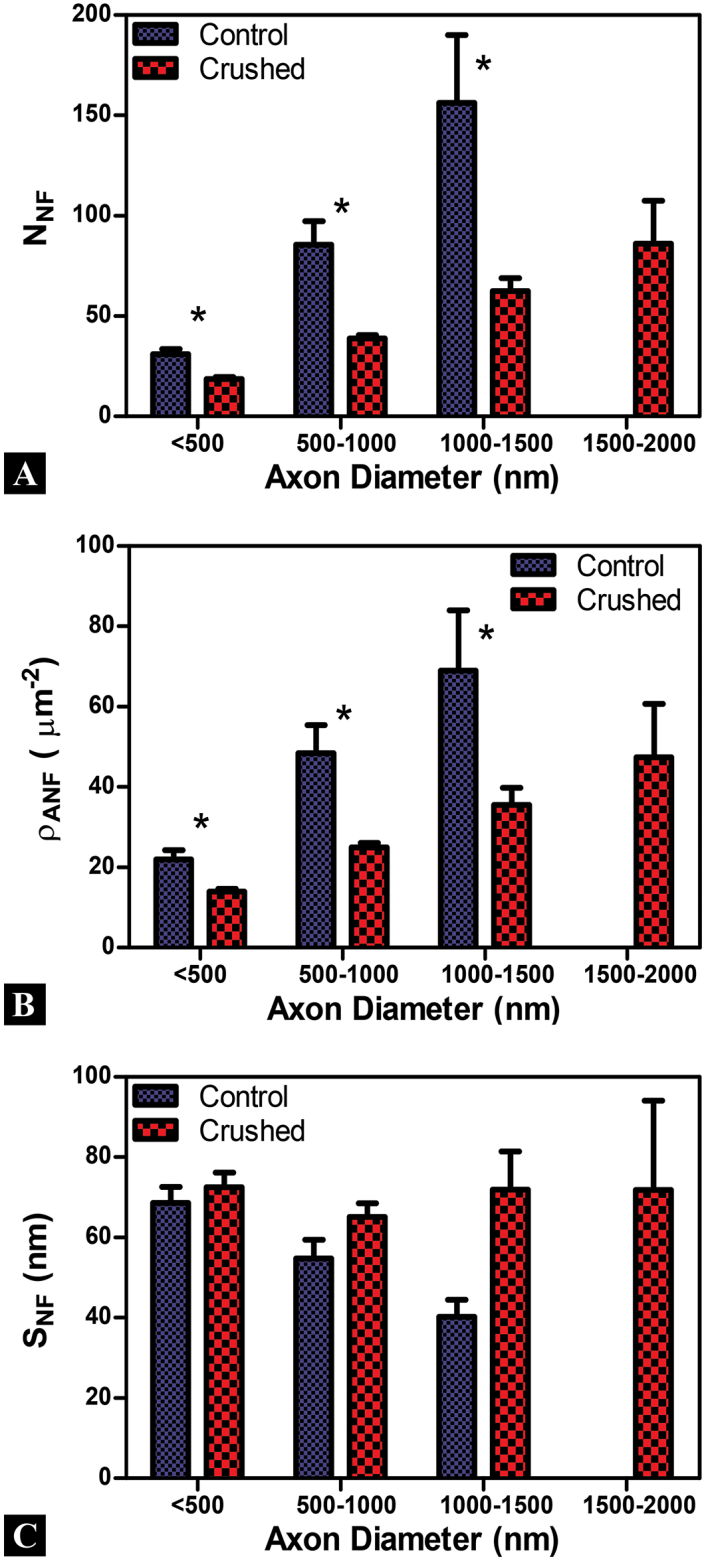
Examination of binned neurofilament data. Axon diameter bins are given along the X-axis and error bars are standard error mean for all plots. (A, B) A strong dependency on axon caliber is observed for the number (*N*_*NF*_) and areal density ($\rho_{A_{NF}}$) of Au-nanoparticles for antibodies labeled neurofilaments in Control and Crushed axons. *N*_*NF*_ and $\rho_{A_{NF}}$ are significantly lower for Crushed across all comparable axon calibers. (C) Spacing between Au-nanoparticles (*S*_*NF*_) showed a strong dependency for Control, but not for Crushed axons. *S*_*NF*_ appears larger for Crushed than Control in nearly all axon diameters and remains approximately 70nm for all Crushed axons regardless of axon diameter. The observed differences between Control and Crushed are more apparent at larger axon diameters. *(p<0.05)

The raw data is binned by axon caliber into 500nm demarcations to contrast and compare between Control and Crushed groups for neurofilaments measures (Fig 7). The number of neurofilaments (as inferred from *N*_*NF*_) increases with axon diameter in the Control group and agrees with observations of the Control axon parameters in the literature (Fig 7A) [16]. Similarly the increase in $\rho_{A_{NF}}$ and decrease in the *S*_*NF*_ for Controls as the diameter of the axon increases has been seen by previous researchers exploring tensile mechanisms of TAI (Fig 7B–7C) [13, 15, 16, 21].

The $N_{NF}$ and $\rho_{A_{NF}}$ are significantly lower in the Crushed group across all comparable axon diameters (p<0.05) (Fig 7A–7B). While *S*_*NF*_ appears higher for Crushed groups, there is no statistically significant difference between axons of comparable axon diameters (Fig 7C). It is interesting to note that for the Crushed axons *S*_*NF*_ does not significantly change as axon diameter increases (unlike for Control) (Fig 7C). As expected, the differences between Control and Crushed neurofilament groups are increased at the larger axon diameters for all comparable measures taken.

The mean number of (${\overset{-}{N}}_{NF}$), areal density (${\overset{-}{\rho }}_{A_{NF}}$), and spacing between (${\overset{-}{S}}_{NF}$) Au-nanoparticles are computed for Control and Crushed neurofilament groups for all bin diameters and are shown in Table 1. Measures taken from the literature, where tensile loading of neuronal axons induced TAI, indicate the percent change from Control increases for ${\overset{-}{N}}_{NF}$ and ${\overset{-}{\rho }}_{A_{NF}}$ and decreases in ${\overset{-}{S}}_{NF}$ [13, 15, 16, 21]. This is counter to our observations, where ${\overset{-}{N}}_{NF}$ and ${\overset{-}{\rho }}_{A_{NF}}$ decrease and ${\overset{-}{S}}_{NF}$ increases for this study. We address all of these results in the discussion.

## Discussion

The hallmarks for TAI are nodal bleb formation and systematic degeneration of cytoskeletal structures along the axon. Using a controlled mechanical environment (the AIM device) with relevant applied loading rates and loads, we find appreciable changes in cytoskeletal spatial distributions within neuronal axons fixed less than 1min after compressive loading. These changes include decreases in the number and density of microtubules and neurofilaments, as well as increases in their spacing, following loading. Our findings suggest that early changes in the neurofilament structure, through sidearm removal or conformational shape change, may serve as a trigger for further secondary damage to the axon, representing a key insight into the temporal aspects of cytoskeletal degeneration at the component level. Another unique result from our study comes from examining the dependency microtubule and neurofilament measures have on axon caliber. This dependence continues to exist immediately following load for most measures (the exception is neurofilament spacing) though the slope of these measures is modified in all cases. Finally the raw data in our study provides evidence that classical approaches of binning axon caliber linked cytoskeletal measures might mask mathematical methods of understanding cytoskeletal distributions.

Data from this study provides measurable insights into cytoskeletal changes <1min after loading. Previous literature has focused on quantifying the spatial distributions of microtubules and neurofilaments as early as 15min after loading. Our data indicates metrics for neurofilament number and density are changing at higher rates than microtubules for the same time period. The literature, which focuses on later time points following injury, provides convincing evidence that microtubules are disrupted; however, little is known of early changes in structural components [13, 15, 16, 21]. Our previous work conducted using confocal imaging demonstrated changes in cytoskeletal expression *in vitro* and *in situ* using the same testing platform [32]. In that study, neurofilaments exhibited a significant 24% decrease in measurable intensity while under load; yet microtubule changes were negligible. While there is a great deal of evidence to support microtubule disruption as a primary mechanism for nodal bleb formation and eventual axon degeneration, our data indicates changes in neurofilaments are preceding measurable spatial changes in microtubules. Variances in the cytoskeletal response between microtubules and neurofilaments to loading may arise from energy dissipation differences. For shorter time frames where fixation occurs within 1min of loading, local intracellular mechanisms may govern the whole cell response. Microtubules, being the most robust component of the cytoskeleton, can reasonably be assumed to not readily degenerate in response to an applied load as compared to less rigid cytoskeletal components like neurofilaments [39]. Thus the simplest cytoskeletal structures within the axon appear to fail before observable changes can be detected for the more rigid constructs.

One goal of this study is to find measurable changes in cytoskeletal distribution and to link these quantifiable metrics to TAI. Previous researchers have indicated the number, spacing, and density of microtubules and neurofilaments in control axons to be measures with a strong dependency on axon caliber [13, 15, 16, 21]. Our Control group data supports this as we observe a strong dependency of *N*_*MT*_ and *N*_*NF*_ on axon caliber. Additionally we observe a larger increase in *N*_*NF*_ than *N*_*MT*_ for increases in axon diameter of Controls, a trend supported by previous researchers [16]. Decreased $\rho_{L_{MT}}$ and increased *S*_*MT*_ has been linked with increasing axon caliber by previous researchers for Controls and is reflected in our data set [13, 15, 16, 21]. Researchers also report increases in *S*_*NF*_ and decreases in $\rho_{A_{NF}}$ with increasing axon diameter, findings we observe for Control axons [15, 16, 21].

Following loading, Crushed axons exhibited significant changes in the measures used, though the axon caliber dependency persisted for most. Our results for Crushed microtubules were supported by previous researchers, but were found to be in contrast for Crushed neurofilaments [13, 15, 16, 21]. The discrepancy between our measured cytoskeletal trends and other observations in the literature may be due to variations in loading as well as temporal differences between our studies. Our study has a focal compressive loading methodology whereas much of the literature uses stretch techniques that are known to produce tensile loading [13, 15, 16, 21]. Temporal differences may also arise because our work focuses on the immediate response of the cytoskeleton to loading (fixation time <1min from loading), whereas previous researchers have quantified their data at later time periods with fixation times ranging between 15min-7days after loading [13, 15, 16, 21].

One explanation for the observed changes in cytoskeletal components is the constituents are displaced into a plane above or below the TEM sections examined. To address this, serial sections were examined and transverse sections were taken. Previous researchers have noted the heterogeneous distribution of cytoskeletal components observed in transverse sections of TEM axon samples [15, 38]. Given this and the loading methodology employed, the present study examined the longitudinal section of the entire length of axons under the compression pad.

The observed drop in the number of microtubules at larger axon calibers for Crushed axons could indicate the axon caliber is increased by the applied load and artificially increases the axon caliber for the associated cytoskeletal metrics. This limitation is supported by the lack of Controls for neurofilament at the axon caliber of 1.5–2.0μm, yet a Crushed population is observed for the same caliber.

An interesting question that arises from the spatial data is the mechanism governing *S*_*NF*_ and why the established relationship with axon caliber appears nullified in Crushed axons. This spacing is mediated by phosphorylation of carboxy-terminals of NF-medium and NF-heavy sidearms where, increases in the number of neurofilaments and carboxy-terminal phosphorylation increase axon caliber [40]. Previous research has indicated that a change in this phosphorylation state, as initiated by applied loads, may lead to alterations in ionic concentrations [5, 41]. The altered homeostasis includes interactions between phosphates and protein kinases and leads to dephosphorylation of the carboxy-terminal resulting in changes to the sidearm structure [5, 41, 42]. In a previous study, using the same experimental model, we hypothesized changes in the protein folding for the neurofilament sidearm structure following loading led to local modifications in conformational shape and spatial distributions within the axon [32]. However, the short time span between loading and fixation of the axon suggests the applied load directly removed the sidearms through a shearing mechanism described by previous researchers who induced axonal degeneration in swine using inertial loading [43].

We posit that local modifications in conformation where NF-medium antibodies label are no longer accessible, due to sidearm removal, result in a decrease of observed Au-nanoparticles. Within the Crushed axon, local modifications to neurofilament sidearms result in a spacing mechanism where a baseline level of approximately 70nm exists regardless of axon caliber. Remarkably the 70nm value we observe for Crushed axon neurofilament spacing has been observed by researchers at later time periods following stretch loading [16, 21].

Further research is needed to examine the early biochemical pathways initiated by neurofilament disruption, and how these disruptions affect microtubule functions over short time durations following loading (<15min). The mechanics of the applied load likely influence the whole cell response resulting TAI. Strain and strain rate, for example, have been implicated by several research groups for governing cytoskeletal responses to applied load [44, 45]. An approach varying the strain rate for focal compression on neuronal axons might provide insights to the rate dependent response of cytoskeletal constituents.

## Conclusion

The present study suggests changes in cytoskeletal spatial distribution can be captured within 1min of loading and the quantification of these distributions can provide insight regarding the temporal evolution of neuronal axons undergoing TAI. The raw data indicates a wealth of information regarding mathematical approaches to understanding cytoskeletal distributions is potentially being ignored by the classical approaches of binning data and insights might be developed by approaching the data in a more open format. The raw data results demonstrate that measurable changes in neurofilament distribution occur ahead of distinguishable changes in microtubule distribution. The results indicate microtubule and neurofilament spatial distribution dependencies on axon caliber continue to occur following applied loads inducing TAI, with the exception of neurofilament spacing. These metrics provide a pathway for connecting changes in cytoskeletal spatial distributions to previously observed changes in measured intensity using confocal microscopy with the same loading platform *in situ* and *in vitro* and may be critical in understanding mechanical failure and degeneration of the cytoskeletal system for neuronal axons undergoing TAI.

## Supporting Information

S1 Arrive Checklist NC3Rs Arrive Checklist. (PDF)

S1 Table Power law fitting coefficients (Eq 4) for microtubule measures of Control and Crushed axons and the 95% confidence intervals for those coefficients. (DOCX)

S2 Table Linear fitting coefficients (Eq 7) for neurofilament measures of Control and Crushed axons and the 95% confidence intervals for those coefficients. (DOCX)
