# Supplementary material for: Changes in Neurofilament and Microtubule Distribution following Focal Axon Compression
Source: PLoS One. 2015 Jun 25;10(6):e0131617. doi: 10.1371/journal.pone.0131617 (PMC4482325; doi:10.1371/journal.pone.0131617)
Supplement: S1 Table — (DOCX) [file pone.0131617.s002.docx]

**S1 Table. Power law fitting coefficients (Equation 4) for microtubule measures of Control and Crushed axons and the 95% confidence intervals for those coefficients.**

| Cytoskeletal Measurement | Load Status | a | (Lower/Upper) 95%  Confidence Interval | m | (Lower/Upper) 95%  Confidence Interval |
| --- | --- | --- | --- | --- | --- |
| MT Number $\boldsymbol{N}_{\boldsymbol{MT}}$ | Control | 0.2173 | (0.1479/0.2867) | 0.5589 | (0.5105/0.6073) |
|  | Crushed | 0.5829 | (0.4279/0.7380) | 0.3523 | (0.3085/0.3962) |
| MT Linear Density $\boldsymbol{\rho}_{\boldsymbol{L}_{\boldsymbol{MT}}}$ (μm^-1^) | Control | 0.1640 | (0.1219/0.2062) | -0.3973 | (-0.4388/-0.3558) |
|  | Crushed | 0.2281 | (0.1611/0.2951) | -0.4838 | (-0.5366/-0.4309) |
| MT Spacing $\boldsymbol{S}_{\boldsymbol{MT}}$ (nm) | Control | 0.7282 | (0.2830/1.173) | 0.6786 | (0.5866/0.7707) |
|  | Crushed | 0.1704 | (0.07747/0.2632) | 0.9932 | (0.9105/1.076) |
