# Supplementary material for: Changes in Neurofilament and Microtubule Distribution following Focal Axon Compression
Source: PLoS One. 2015 Jun 25;10(6):e0131617. doi: 10.1371/journal.pone.0131617 (PMC4482325; doi:10.1371/journal.pone.0131617)
Supplement: S2 Table — (DOCX) [file pone.0131617.s003.docx]

**S2 Table. Linear fitting coefficients (Equation 8) for neurofilament measures of Control and Crushed axons and the 95% confidence intervals for those coefficients.**

| Cytoskeletal Measurement | Load Status | $\boldsymbol{p}_{\boldsymbol{1}}$ | (Lower/Upper) 95%  Confidence Interval | $\boldsymbol{p}_{\boldsymbol{2}}$ | (Lower/Upper) 95%  Confidence Interval |
| --- | --- | --- | --- | --- | --- |
| NF Number $\boldsymbol{N}_{\boldsymbol{NF}}$ | Control | 0.1357 | (0.1070/0.1645) | -5.727 | (-17.34/5.883) |
|  | Crushed | 0.05205 | (0.04571/0.05839) | 2.204 | (-1.934/6.343) |
| NF Areal Density $\boldsymbol{\rho}_{\boldsymbol{A}_{\boldsymbol{NF}}}$ (μm^-2^) | Control | 0.0595 | (0.0377/0.08129) | 6.115 | (-2.689/14.92) |
|  | Crushed | 0.02663 | (0.02663/0.03099) | 5.659 | (-2.819/8.5) |
| NF Spacing $\boldsymbol{S}_{\boldsymbol{NF}}$ (nm) | Control | -0.01985 | (-0.05361/0.01392) | 72.89 | (59.25/86.52) |
|  | Crushed | -0.004766 | (-0.01966/0.01013) | 72.22 | (62.5/81.95) |
